# Gene expression profiling for the diagnosis of male breast cancer

**DOI:** 10.1186/s12885-024-13358-4

**Published:** 2024-12-27

**Authors:** Jing Liu, Yifeng Sun, Peng Qi, Yixin Wo, Yue Pang, Qinghua Xu, Midie Xu, Shenglin Huang, Qifeng Wang

**Affiliations:** 1 Department of Pathology, Fudan University Shanghai Cancer Center, and Shanghai Key Laboratory of Medical Epigenetics, International Co-laboratory of Medical Epigenetics and Metabolism, Institutes of Biomedical Sciences, Shanghai Medical College, Fudan University, Shanghai, China; 2 Canhelp Genomics Research Center, Canhelp Genomics Co., Ltd, Hangzhou, China

**Keywords:** Male breast cancer, Gene expression profile, Tissue of origin

## Abstract

**Background:**

Male breast cancer (MBC) is a rare malignancy, but its global incidence has shown a notable increase in recent decades. Factors such as limited health literacy, inadequate health education, and reluctance to seek medical attention contribute to the late-stage diagnosis of most MBC patients. Consequently, there is an urgent need for a highly specific and sensitive diagnostic approach to MBC.

**Methods:**

This retrospective study enrolled 20 patients with 30 surgical or biopsy MBC specimens from August 2020 to August 2023. The 90-gene expression assay was performed to determine the tissue of origin. Predicted tumor types were then compared to the reference diagnosis for accuracy calculation. The differentially expressed genes were identified between male and female breast cancer.

**Result:**

The 90-gene expression assay demonstrated an overall accuracy of 96.7% (29/30) when compared with the pathological diagnosis. For primary, lymph node metastatic, and distant metastatic tumors, the accuracies were 100% (15/15), 90.9% (10/11), and 100% (4/4), respectively. Five genes (*RPS4Y1*,* PI15*,* AZGP1*,* PRRX1*, and *AGR2*) were up-regulated, and six (*XIST*,* PIGR*,* SFRP1*,* PLA2G2A*,* S100A2*, and *CHI3L1*) were down-regulated in MBC.

**Conclusion:**

Our findings highlight the promising performance of the 90-gene expression assay in accurately identifying the tumor origin in MBC. Incorporating this assay into pathological diagnoses has the potential to empower oncologists with precision treatment options, ultimately enhancing the care and outcomes for patients with MBC.

**Supplementary Information:**

The online version contains supplementary material available at 10.1186/s12885-024-13358-4.

## Background

Male breast cancer (MBC) is a rare malignancy, accounting for less than 1% of all breast cancer cases but its global incidence has increased over recent decades [1, 2]. The etiology and pathogenesis of MBC remain unclear. Studies have shown that risk factors for MBC include a family history of breast cancer, older age, carrying a predisposition germline genetic mutation (e.g., *BRCA 1/2*), hormonal imbalance, and exposure to radiation [3]. Owing to factors such as illiteracy, inadequate health education, and reluctance to report symptoms to the clinical physician, most MBC patients present at an advanced stage [4]. A study conducted at a single institution in Italy reported distant metastasis in 32% of MBC patients, primarily affecting the lungs and bones [5]. Consequently, precise diagnosis of MBC is essential for selecting appropriate treatment and improving prognosis.

Male breast tissue is typically less developed than its female counterpart, rendering physical examination and imaging interpretation more challenging [6]. Both mammography and magnetic resonance imaging have limited diagnostic value in MBC [7, 8]. Histopathology is considered the gold standard for diagnosing MBC metastases. Various immunohistochemical markers including androgen receptor (AR), GATA3, mammaglobin (MGB), and gross cystic disease fluid protein-15 (GCDFP15), play a crucial role in diagnosing the majority of metastatic MBC [1]. However, due to the rarity of MBC and the lack of awareness among both patients and healthcare professionals, misdiagnosis or delayed diagnosis of MBC is prevalent [9]. Thus, identifying the tissue of origin of metastatic MBC, especially in patients without a prior history of breast cancer, is challenging. Furthermore, survivors of MBC face elevated risks of developing second primary cancers [10]. Accurate diagnosis of second primary cancers or MBC metastasis is crucial for selecting appropriate therapy.

As the field of oncology continues to evolve towards precision medicine including targeted therapy, immune checkpoint inhibitors, chimeric antigen receptor T cell therapy, and cancer vaccines, etc., the integration of molecular diagnostics has become increasingly important [11–13]. Previously, our research group developed a 90-gene expression assay (Canhelp-Origin Test) to identify 21 common tumor types using real-time PCR methods with total RNA isolated from formalin-fixed, paraffin-embedded (FFPE) tumor tissue [14]. In a large-scale multicenter study comprising 1417 samples, the 90-gene expression assay demonstrated an accuracy of 94.4% and a specificity exceeding 99% [15]. A randomized phase III trial demonstrated that site-specific treatment guided by the 90-gene-expression assay resulted in more therapy options, significantly improved progression free survival and favorable overall survival compared with empirical chemotherapy in patients with cancer of unknown primary [16]. In the present study, we conducted a retrospective study to assess the performance of the 90-gene expression assay and explore its potential diagnostic utility for MBC.

## Materials and methods

### Patient enrollment and specimen acquisition

The study was conducted with approval from the institutional review board of Fudan University Shanghai Cancer Center (FUSCC, Shanghai, China). We collected surgical or biopsy specimens, along with associated clinicopathologic data. Inclusion criteria were: (1) FFPE tissues, (2) the reference diagnosis was male breast cancer (3) more than 60% tumor cell content, and less than 40% necrosis as assessed by Hematoxylin and Eosin (H&E) staining [15].

### Clinicopathological characteristics

Patient demographics including age, tumor site, tumor dimensions, lymph node involvement, and surgical approach were meticulously documented from medical records. Tumor staging was conducted according to the eighth edition of the American Joint Committee on Cancer (AJCC) staging system [17]. Diagnoses were confirmed through an independent review of all case slides by two pathologists (QF W and J L). Histological types were characterized according to the World Health Organization (WHO) guidelines [18], while histological grading for invasive cancers was defined using a modified Bloom and Richardson scoring scheme [19]. Immunohistochemical analyses were performed on 4 μm sections of FFPE tissues, with a detailed list of all antibodies utilized provided in Table S1.

### Sample preparation and RNA isolation

A range of five to fifteen unstained sections, each 5 μm thick, were prepared and centralized for analysis. Pathologists QF W and J L reviewed H&E-stained slides to assess the tumor cell content, identify tumor regions, and manually perform macro-dissection for enrichment. Adhering to established protocols, total RNA was extracted utilizing the FFPE total RNA Isolation Kit (Canhelp Genomics Co., Ltd., Hangzhou, China) [14]. Subsequently, the concentration and purity were measured using a spectrophotometer.

### Gene expression profiling analysis

The 90-gene expression assay (Canhelp Genomics Co., Ltd) was executed in adherence to established protocols, encompassing reverse transcription of total RNA and real-time PCR reaction for comprehensive tumor-specific gene expression profiling [14, 15, 20]. The results of the 90-gene expression assay were reported if they met the PCR analytical quality control threshold for internal controls (cycle threshold value less than 38) and no template control (cycle threshold value larger than 38) [20]. For each case, the prespecified 90-gene classifier was utilized to analyze the gene expression patterns and generate similarity scores for every primary tumor type. These scores are probability-based, ranging from 0 to 100, and the total of all 21 similarity scores for each sample sums to 100. The tumor type with the highest similarity score was considered the predicted type.

### Statistical analysis

Statistical analyses were conducted using R software (version 3.6.1) and Bioconductor packages (version 3.9). Hierarchical clustering of specimens based on gene expression profiling was performed with the “pheatmap” package (version 1.0.12). Differentially expressed genes between MBC and FBC were identified using the “limma” package (version 3.54.2) with Benjamini-Hochberg (B-H) p-value < 0.01.

## Results

### Patients and samples

Initially, 20 patients archived from August 2020 and August 2023 were enrolled in this study, yielding 30 FFPE specimens, comprising 15 primary breast tumors (PBT), 11 lymph node metastases (LNM), and 4 distant organ metastases (DOM). Table 1 displays the clinicopathological characteristics of 20 patients. The median age at diagnosis was 65.5 years for all patients. Among the PBT cases, 10 occurred in the left breast and 5 in the right. Eight tumors measured less than 2 cm, and seven ranged between 2 and 5 cm. All histological subtypes were identified as invasive ductal carcinoma, with two cases exhibiting invasive ductal carcinoma coexisting with invasive micropapillary carcinoma. Surgical interventions comprised modified radical mastectomy (*N* = 11), simple mastectomy (*N* = 3), and simple mastectomy with sentinel lymph node biopsy (*N* = 1). Histologically, 12 (80%) PBTs were classified as Grade 2, and 3 (20%) were classified as Grade 3. Table 2 provides detailed clinical and immunohistochemistry (IHC) staining features of MBC. For each case, clinical parameters such as age, tumor size (in cm), and pathological staging are presented. The IHC results indicated positive staining for estrogen receptor (ER) (18/19, 94.7%), progesterone receptor (PR) (17/20, 85.0%), GATA3 (20/20, 100%), HER2 (3/20, 15.0%), AR (17/19, 89.5%), GCDFP15 (16/18, 88.9%), TRPS1 (13/13, 100%), and MGB (2/4, 50.0%).

**Table 1:** Patient’s general characteristics included in this study

Characteristic	Primary tumor/paired lymph node metastasis	Distant organ metastasis
No. patients	15/11	4
Age (years)	65.5 (27 ∼ 86)	
Pathological type		
IDC	13/10	4
IDC with invasive micropapillary	2/1	0
Tumor site		
Left	10/7	/
Right	5/4	/
Tumor size (cm)		
≤ 2	8	/
2–5	7	/
Surgical approach		
Modified mastectomy	11	
Simple mastectomy with SLN	1	
Simple mastectomy	3	
Histological grade		
1	0	/
2	12	/
3	3	/
Metastatic sites		
Lung	/	2
Bone	/	1
Thoracic wall	/	1

IDC: Invasive ductal carcinoma; SLN: Sentinel lymph node; /: Censored value

**Table 2:** Clinical information and immunohistochemistry staining features of male breast cancer

no.	Type	Age	Size (cm)	pT	pN	M	ER	PR	HER2 (IHC)	HER2 (FISH)	GATA3	AR	GCDFP15	TRPS1	MGB
1/2	PBT/LNM	27	5	2	1a	0	+	+	2 +	-	+	+	+	+	/
3/4	PBT/LNM	67	2.5	2	1a	0	+	+	1 +	/	+	+	+	+	/
5/6	PBT/LNM	68	1	1b	2a	0	+	+	1 +	/	+	+	+	+	/
7/8	PBT/LNM	70	3	2	1a	0	+	+	1+	/	+	-	+	+	/
9/10	PBT/LNM	73	2	1c	1a	0	+	+	1+	-	+	+	+	+	/
11/12	PBT/LNM	44	1.1	1c	1a	0	+	+	1+	/	+	+	+	+	/
13/14	PBT/LNM	63	2.5	2	3a	0	+	+	2+	+	+	+	+	/	/
15/16	PBT/LNM	69	1.8	1c	2a	0	+	+	0	/	+	-	+	/	-
17/18	PBT/LNM	57	3	2	2a	0	-	-	3+	/	+	+	+	/	/
19/20	PBT/LNM	86	2	1c	3a	0	+	+	2+	-	+	+	+	/	/
21	PBT	52	3.2	2	0	0	+	+	1+	/	+	+	-	+	/
22	PBT	60	1.3	1c	0	0	+	+	1+	/	+	+	-	+	/
23	PBT	70	3	2	0	0	+	+	2+	-	+	+	+	+	/
24	PBT	67	2	1c	0	0	+	+	0	/	+	+	+	+	/
25	PBT	67	1.5	1c	0	0	+	+	1+	-	+	+	+	+	/
26	LNM	74	/	/	/	/	+	+	1+	/	+	+	+	+	+
27	DOM	44	/	/	/	/	+	+	0	/	+	/	/	+	/
28	DOM	62	/	/	/	/	+	+	1+	/	+	+	/	/	/
29	DOM	64	/	/	/	/	+	-	1+	/	+	+	+	/	-
30	DOM	53	/	/	/	/	/	-	3+	/	+	+	+	/	+

PBT: Primary tumor; LNM: Lymph node metastasis; DOM: Distant organ metastasis

### Performance of the 90-gene expression assay in male breast cancer

Total RNA was isolated from FFPE sections of 30 samples. Concentrations ranged from 9.7 to 671.1 ng/µL, with a median concentration of 224.5 ng/µL. The A260/A280 ratio ranged from 1.84 to 1.95. All specimens met all quality control criteria and were successfully analyzed using the 90-gene expression assay.

**Fig. 1:**
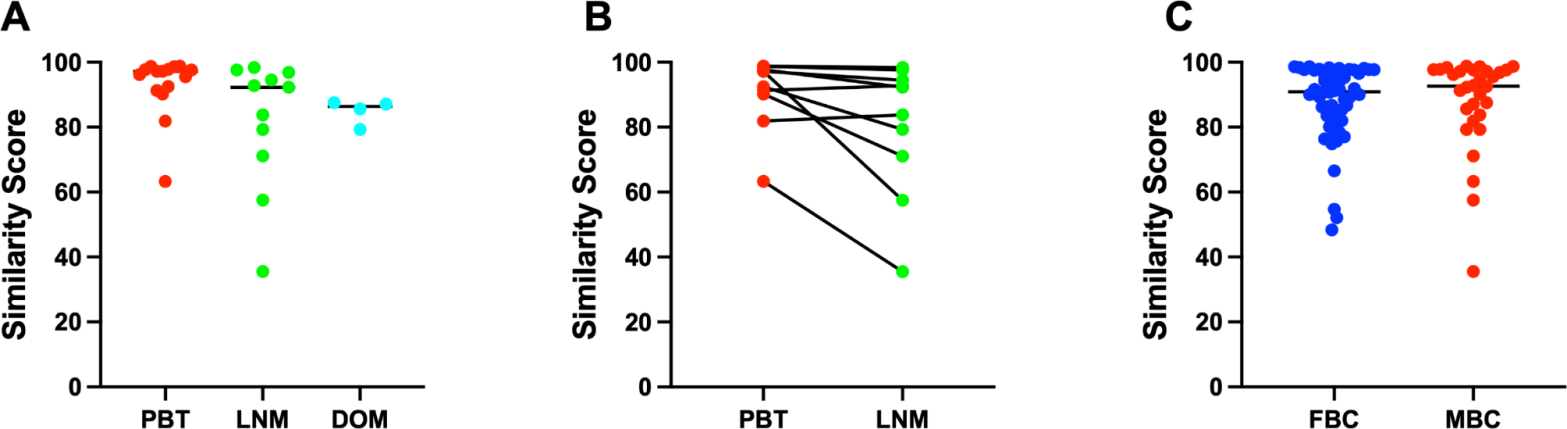
**(A)** The distribution of similarity score for the PBT (red dots), LNM (green dots), and DOM (cyan dots), **(B)** The similarity score of 10 paired PBT and LNM samples, **(C)** The distribution of similarity score for the female (blue dots) and male (red dots) breast cancer

The results showed an overall concordance rate of 96.7% (29/30) with the reference diagnoses. Specifically, the consistency rates were 100% (15/15) for PBT cases, 90.9% (10/11) for LNM cases, and 100% (4/4) for DOM cases (Table 3). The median similarity scores generated by the 90-gene expression assay were 97.2 (range 63.3 ∼ 98.8) for PBT, 92.3 (range 57.5 ∼ 98.4) for LNM, and 86.4 (range 79.3 ∼ 87.5) for DOM (Fig. 1A). The differences in the similarity scores between paired specimens (PBT and LNM) are depicted in Fig. 1B.

**Table 3:** Performance of 90-gene expression assay in male breast cancer

Tumor type	*n*	Agreement	Accuracy (%)
Primary male breast cancer	15	15	100
Lymph node metastasis	11	10	90.9
Distant organ metastasis	4	4	100
Total accuracy	30	29	96.7

### Comparison of the gene expression profiling between male and female breast cancer

**Fig. 2:**
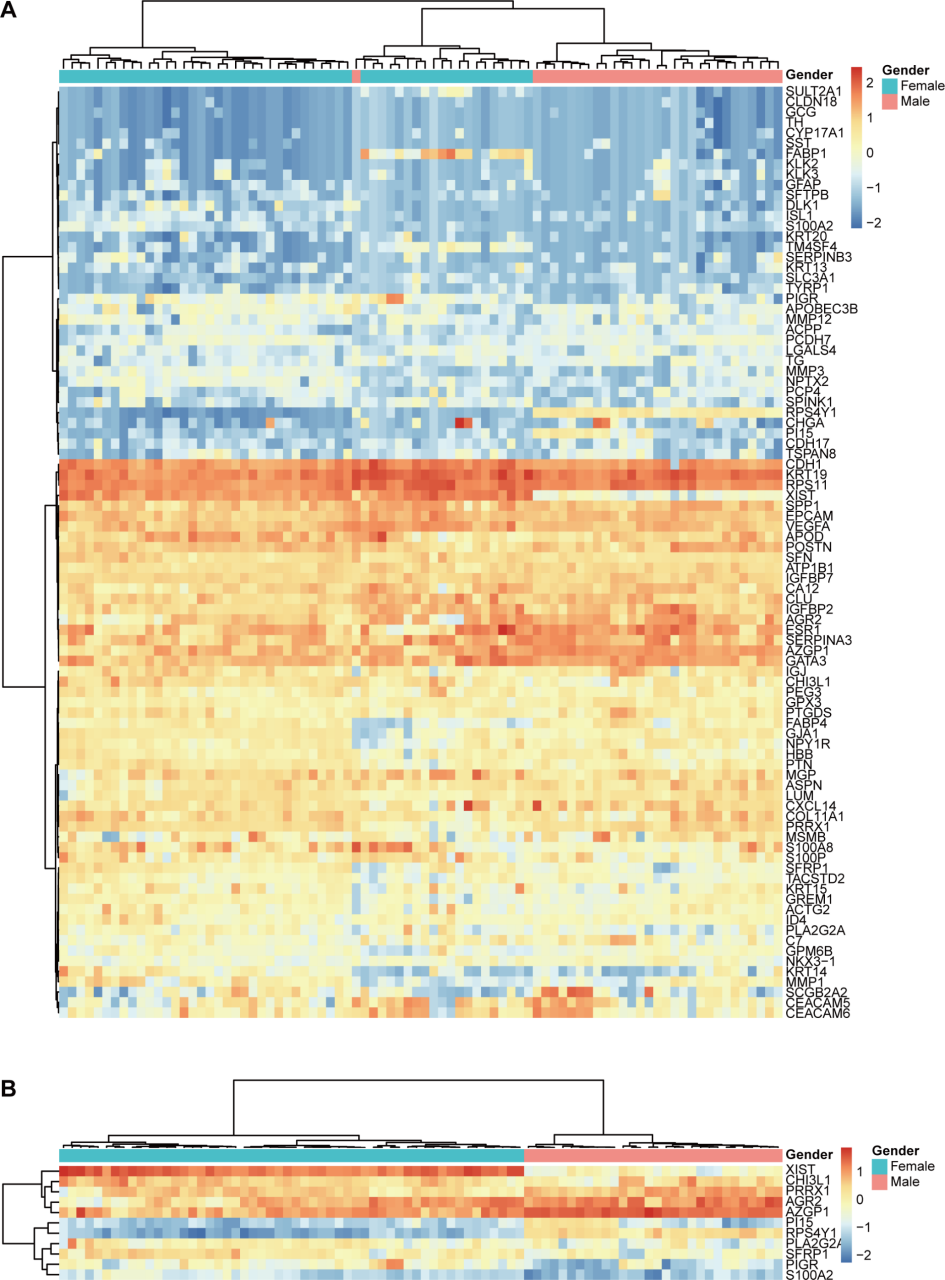
Hierarchical clustering analysis was conducted on **(A)** 90 genes and **(B)** 11 differentially expressed genes across 84 specimens. The left panel displays a dendrogram depicting the hierarchical clustering of genes. Colored pixels represent the magnitude of the gene expression intensities, with shades of red and blue indicating over-expression and under-expression, respectively, relative to the mean for each gene. The upper panel shows a dendrogram illustrating the hierarchical clustering of samples. The upper panel indicates clinical features including the gender of each sample

This study further included 54 cases of female breast cancer (FBC) for comparison of gene expression profiling with MBC. These specimens included 19 cases of metastatic breast cancer from the study by Wang et al. [21] and 35 cases of primary breast cancer from the study by Sun et al. [15]. The distribution of similarity scores for MBC (median 92.7, range 35.5–98.8) and FBC (median 90.9, range 48.4–98.6) is shown in Fig. 1C. Hierarchical clustering based on the 90 genes revealed a distinct separation between 30 cases of MBC and 54 cases of FBC **(**Fig. 2A**)**.

Furthermore, differentially expressed genes were identified from the 90-gene panel comparing MBC and FBC, with B-H adjusted p-value below 0.01. Among them, five genes (*RPS4Y1*,* PI15*,* AZGP1*,* PRRX1*, and *AGR2*) were up-regulated, and six genes (*XIST*,* PIGR*,* SFRP1*,* PLA2G2A*,* S100A2*, and *CHI3L1*) were down-regulated in MBC **(**Fig. 2B**)**. Further details about these genes are described in Table 4.

**Table 4:** Desription of 5 up-regulated and 6 down-regulated genes in male breast cancer

Symbol	Description	Cytoband	*P*-value	adjusted *P*-value	Regulation (MBC/FBC)
RPS4Y1	Ribosomal Protein S4 Y-Linked 1	Yp11.2	3.09E-49	2.78E-47	Up
PI15	Peptidase Inhibitor 15	8q21.13	1.73E-08	5.18E-07	Up
AZGP1	Alpha-2-glycoprotein 1, zinc-binding	7q22.1	1.96E-07	4.41E-06	Up
PRRX1	Paired Related Homeobox 1	1q24.2	2.73E-04	3.51E-03	Up
AGR2	Anterior Gradient 2	7p21.1	7.44E-04	6.09E-03	Up
XIST	X Inactive Specific Transcript	Xq13.2	1.04E-42	4.66E-41	Down
PIGR	Polymeric immunoglobulin receptor	1q31-q41	4.30E-07	7.73E-06	Down
SFRP1	Secreted Frizzled Related Protein 1	8p11.21	6.64E-07	9.95E-06	Down
PLA2G2A	Phospholipase A2 Group IIA	1p36.13	4.72E-04	4.54E-03	Down
S100A2	S100 Calcium Binding Protein A2	1q21.3	4.88E-04	4.54E-03	Down
CHI3L1	Chitinase 3 Like 1	1q32.1	5.05E-04	4.54E-03	Down

### Specific case

Among all cases, only one lymph node metastasis specimen (Sample 16) was misclassified by the 90-gene expression assay, incorrectly identifying breast cancer as a neuroendocrine tumor. The top two results were neuroendocrine tumor with a similarity score of 54.6 and breast cancer with a similarity score of 35.5. The predicted result indicated it was a neuroendocrine tumor, which was inconsistent with the reference diagnosis.

We further reviewed the clinicopathological information and performed additional IHC staining for neuroendocrine markers. This patient is a 69-year-old male who underwent seven months of tamoxifen neoadjuvant therapy followed by modified radical mastectomy. Microscopically, we observed eosinophilic change in tumor cell cytoplasm, tumor stroma fibrosis, and morphological features of locally metastatic lymph nodes showing neuroendocrine differentiation, along with post-treatment response. IHC markers including synaptophysin (Syn), chromogranin A (CgA), and INSM1 were all positive, consistent with the findings of the 90-gene expression assay. Finally, this case is diagnosed as male breast cancer with neuroendocrine differentiation. The histological morphology and IHC results of this case are depicted in Figure S1.

## Discussion

This study evaluated the potential diagnostic utility of the 90-gene expression assay for diagnosing MBC. The overall agreement of 96.7% (29/30) indicates the excellent performance of the 90-gene expression assay in identifying the tumor of origin in primary or metastatic MBC. The subgroup analysis shows consistency rates of 100% for PBT and DOM cases and 90.9% for LNM cases.

In our previous study, we conducted two multicenter validation studies to evaluate the performance of the 90-gene expression assay in FBC. The results demonstrated accuracies of 97.8% (44/45) and 98.4% (121/123), respectively [14, 15]. Additionally, Qifeng et al. also validated the 90-gene expression assay in primary and metastatic triple-negative breast cancer among females, showing an overall accuracy of 97.4% (112/115) [22]. Therefore, the results of all these studies in females are consistent with the present study’s findings in males.

Shaaban et al. compared the IHC patterns of MBC and FBC and reported that the proportion of ER-positive cases were significantly higher in MBC compared to FBC (80% vs. 68%), while PR-positive rates were similar (71% vs. 72%) [23]. In our study, we observed higher ER and PR positivity rates, at 94.7% (18/19) and 85% (17/20), respectively, and a HER2 positivity of 15% (3/20). In addition to IHC-based findings, Callari et al. and Johansson et al. performed comprehensive analyses of gene expression patterns in MBC and FBC, identifying significant differences that underscore the notion that MBC and FBC are distinct diseases [24, 25]. Our study aligns with these findings, revealing significant differential expression of 11 genes in MBC compared to FBC using the 90-gene expression assay. Specifically, five genes were up-regulated, and six genes were down-regulated in MBC. The *RPS4Y1* and *XIST* genes are located on sex chromosomes. Yuxi et al. investigated that *XIST*, a long noncoding RNA, has a role in promoting breast cancer stem cell self-renewal by derepressing let-7 controlled paracrine IL-6 proinflammatory signaling [26]. *AZGP1* has been recognized as a crucial promoter in cancer metastasis and lipid metabolism [27]. Higher expression of *AZGP1* in T cells has also been observed in MBC, as reported by Handong et al., suggesting the immunological and metabolic differences between MBC and FBC [28]. Several studies have noted correlations between *PI15*,* PRRX1*,* AGR2*,* PIGR*,* SFRP1*,* PLA2G2A*,* S100A2*, and *CHI3L1* genes and breast cancer, but have not elucidated their differential expression in MBC and FBC [29–36]. The diagnostic significance of these findings is particularly noteworthy. The differential expression of these genes may serve as potential biomarkers for understanding its unique metastatic behavior and guiding treatment approaches. Due to the rarity of MBC, researches on its molecular aspects are currently limited, but our findings provide valuable insights into its biological characteristics and clinical relevance. These results not only confirm the distinct molecular profiles of MBC and FBC but also underscore the need for further research to translate these findings into personalized diagnostic and therapeutic strategies.

This study also had several limitations. First, cases of MBC with neuroendocrine differentiation might be misclassified as a neuroendocrine tumor rather than breast cancer. Second, the gene expression analysis was limited to 90 genes, which constrains the exploration of the broader molecular landscape. Future studies integrating transcriptomic sequencing and single-cell analysis could deeper insights into the molecular mechanisms underlying MBC [37]. Additionally, exploring the role of ion channels, such as voltage-gated sodium channels (VGSCs) and transient receptor potential melastatin 7 (TRPM7), represents a promising avenue for identifying novel therapeutic targets in MBC [38, 39].

## Conclusions

In conclusion, the 90-gene expression assay demonstrates high accuracy in diagnosing both primary and metastatic MBC, indicating its significant potential as a supplementary diagnostic tool for MBC. Incorporating this assay into pathological diagnoses holds the potential to empower oncologists with precision treatment options, ultimately enhancing the care and outcomes for patients with MBC. Furthermore, several molecular biomarkers may help elucidate the distinct mechanisms underlying MBC and FBC.

## Electronic supplementary material

Below is the link to the electronic supplementary material.

Supplementary Material 1: Table S1. Antibodies characterization for immunostaining

Supplementary Material 2: Fig. S1. The histologic features and immunohistochemical profiles of Sample 16. (A) Neuroendocrine differentiation of cells (200X). The tumor cells were (B) positive for CgA (200X). (C) Diffusely and strongly positive for Syn (200X). (D) Weakly positive for INSM1 (200X)

## Data Availability

The datasets used and/or analysed during the current study are available from the corresponding author on reasonable request.

## Abbreviations

AJCC: American Joint Committee on Cancer
AR: Androgen receptor
DOM: Distant organ metastase
FBC: Female breast cancer
FFPE: Formalin-fixed, paraffin-embedded
FNA: Fine-needle aspiration
FUSCC: Fudan University Shanghai Cancer Center
GCDFP15: Gross cystic disease fluid protein-15
LNM: Lymph node metastase
MBC: Male breast cancer
MGB: Mammaglobin
NCB: Needle core biopsy
PBT: Primary breast tumor
TRPM7: Transient receptor potential melastatin 7
VGSC: Voltage-gated sodium channels
WHO: World Health Organization
