# Supplementary material for: Gene expression profiling for the diagnosis of male breast cancer
Source: BMC Cancer. 2024 Dec 27;24:1584. doi: 10.1186/s12885-024-13358-4 (PMC11681697; doi:10.1186/s12885-024-13358-4)
Supplement: Supplementary file 1 — Supplementary Material 1: Table S1. Antibodies characterization for immunostaining [file 12885_2024_13358_MOESM1_ESM.docx]

***Supplementary Material***

1. **Supplementary Table**

**Supplemental Table 1** Antibodies characterization for immunostaining

| Antibody | Vendor | Clone | Dilution |
| --- | --- | --- | --- |
| ER | ROCHE | SP1 | Using antibody |
| PR | ROCHE | 1E2 | Using antibody |
| HER2 | ROCHE | 4B5 | Using antibody |
| GATA3 | ROCHE | L50-823 | Using antibody |
| AR | DAKO | AR441 | 1:50 |
| GCDFP15 | ROCHE | EP1582Y | Using antibody |
| TRPS1 | DAKO | EPR16171 | Using antibody |
| Mammaglobin | ROCHE | 31A5 | Using antibody |
