# Supplementary figures and images for: Gene expression profiling for the diagnosis of male breast cancer
Source: BMC Cancer. 2024 Dec 27;24:1584. doi: 10.1186/s12885-024-13358-4 (PMC11681697; doi:10.1186/s12885-024-13358-4)

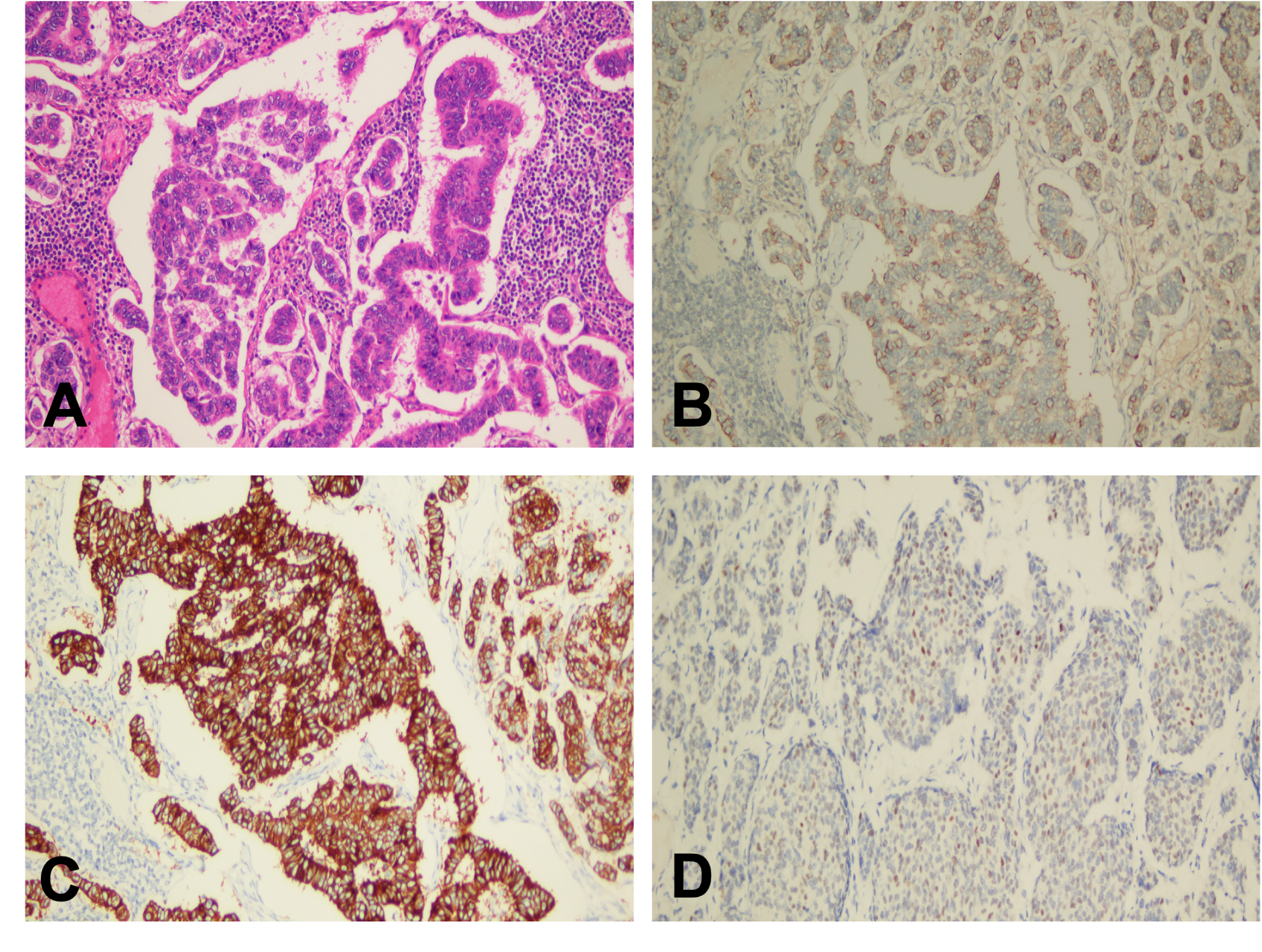

Supplement: Supplementary file 2 — Supplementary Material 2: Fig. S1. The histologic features and immunohistochemical profiles of Sample 16. (A) Neuroendocrine differentiation of cells (200X). The tumor cells were (B) positive for CgA (200X). (C) Diffusely and strongly positive for Syn (200X). (D) Weakly positive for INSM1 (200X) [file 12885_2024_13358_MOESM2_ESM.tif]
